# Comparison between typical primary and eunatraemic, eukalaemic hypoadrenocorticism: 92 cases

**DOI:** 10.1186/s13620-024-00280-1

**Published:** 2024-09-28

**Authors:** Adrien Joaquim Da Silva, Eilidh Gunn, Pedro Jose Guzmán Ramos, Robert Edward Shiel, Laura Bree, Carmel Therese Mooney

**Affiliations:** 1 University College Dublin Veterinary Hospital, Dublin, Ireland; 2 North Downs Specialist referrals, Bletchingley, UK; 3 The Animal Hospital, Murdoch University, Murdoch, Australia; 4 London Veterinary Specialists, London, UK

**Keywords:** Hypoadrenocorticism, Atypical, Glucocorticoid, Aldosterone, Central

## Abstract

**Background:**

Naturally occurring hypoadrenocorticism is an uncommon endocrine disorder in dogs but has significant morbidity and mortality. Some dogs present with apparent glucocorticoid deficiency alone as evidenced by eunatraemia and eukalaemia. Few studies have compared dogs with hypoadrenocorticism with or without electrolyte disturbances and there are no large case series of affected dogs from Ireland.

**Methods:**

Retrospective observational study.

**Results:**

Ninety-two cases diagnosed with hypoadrenocorticism subdivided into those with supportive electrolyte disturbances (Group 1; *n* = 72) and those without (Group 2; *n* = 20). Dogs in Group 1 were significantly (*p* = 0.001) younger (4.0 (3.0–6.0) years) than dogs in Group 2 (6.0 (4.75–8.25) years). Dogs in Group 1 presented significantly more commonly with vomiting (Group 1: 52/71 (73.2%), Group 2: 6/20 (30.0%); *p* < 0.001), total hyperproteinaemia (Group 1: 21/71 (29.6%), Group 2: 1/20 (5.0%); *p* = 0.023), increased urea (Group 1: 52/72 (72.2%), Group 2: 5/20 (25.0%); *p* < 0.001), increased creatinine (Group 1: 31/72 (43.1%), Group 2: 3/20 (15.0%); *p* = 0.021) and hyperphosphataemia (Group 1: 40/71 (56.3%), Group 2: 2/20 (10.0%); *p* < 0.001), and significantly less commonly with reticulocytosis (Group 1: 4/38 (10.5%), Group 2: 5/13 (38.5%), *p* = 0.023). An undetectable basal aldosterone concentration had a positive predictive value of 94.3% for diagnosing undetectable post-ACTH aldosterone concentration. Of the thirteen dogs in Group 2 that had aldosterone concentrations measured and secondary disease excluded, 7 (53.8%) had or subsequently developed evidence of aldosterone deficiency, although not always with electrolyte abnormalities.

**Conclusions:**

Dogs with hypoadrenocorticism from Ireland are similar to other reported cases. An undetectable basal aldosterone concentration is highly predictive of mineralocorticoid deficiency. Dogs with apparent glucocorticoid deficiency alone can progress to more typical disease and should be monitored appropriately.

## Background

Naturally occurring hypoadrenocorticism is considered a relatively uncommon endocrine disorder in dogs with an estimated prevalence of < 0.33% [3, 17, 21]. However, it is an important disease because of its potential for significant morbidity and mortality and tendency towards a higher prevalence in certain breeds. Primary hypoadrenocorticism is considered to account for the majority (> 90%) of cases [28] and is presumed to result from autoimmune destruction as supported by the presence of circulating autoantibodies against cytochrome P450 side-chain cleavage enzyme and histopathological evidence of lymphocytic adrenalitis in some affected dogs [4, 9, 10]. Nevertheless, other pathologies that result in adrenal destruction, including neoplasia, tuberculosis, granulomatous diseases and haemorrhage can occur, albeit rarely [15, 23]. Secondary hypoadrenocorticism, arising from pituitary or hypothalamic disease, is less common in dogs. Where definitive causes of central disease have been investigated, they included pituitary neoplasia, brain trauma and lymphocytic hypophysitis [15].

Since the first large case series describing hypoadrenocorticism in dogs was published, it has been known that not just dogs with secondary disease but also some with primary disease present with evidence of glucocorticoid deficiency alone [28]. These are often called atypical cases or more recently, as suggested by the European Society of Veterinary Endocrinology, eukalaemic, eunatraemic hypoadrenocorticism (EEH) stratified by being primary or secondary, if evaluated. This subpopulation of dogs has become the subject of growing interest. Specific case series have included various numbers of dogs from 7 to 40 [16, 25, 31, 32, 34, 35]. Although these dogs are often considered to be older, with a more protracted history and more commonly afflicted with anaemia, hypoalbuminaemia and hypocholesterolaemia, very few studies have specifically analysed differences statistically [16, 34]. These cases remain a diagnostic challenge as they have no pathognomonic features and require specific assessment to rule in or out hypoadrenocorticism. Such cases may be more common than previously thought with a prevalence of 44.4% (59 of 133 dogs with hypoadrenocorticism) in one recent study [30]. Hypoadrenocorticism is frequently considered a differential diagnosis of chronic gastrointestinal disease where it can account for 0.3 to up to 4% of cases [8, 18, 33]. Given the high prevalence of chronic gastrointestinal disease in primary care and referral practice, it is likely that more cases of EEH could be diagnosed in the future if specifically tested.

Neither the term atypical nor eukalaemic, eunatraemic primary hypoadrenocorticism provide useful information on why dogs with primary disease present without electrolyte abnormalities. Selective destruction of the zonae fasciculata and reticularis with relative sparing of the zona glomerulosa has been described [1, 22]. Symptomatic therapy, particularly intravenous fluids, could potentially mask pre-existing electrolyte abnormalities [28]. Additionally, dogs with known mineralocorticoid deficiency can maintain electrolyte concentrations within reference interval through unknown mechanisms [2]. In all affected cases, progression to an Addisonian crisis is possible although how often and within what timeframe this occurs has not been well evaluated.

There is only one report describing dogs with hypoadrenocorticism presenting to a veterinary practice within the Republic of Ireland but it was focused on the treatment of Addisonian crises and forms a small sub population of the current study [13]. The aim of this study was to describe a larger cohort of dogs from Ireland diagnosed with hypoadrenocorticism. A secondary aim was to investigate any significant differences in signalment, clinical features and clinicopathological abnormalities between those with or without electrolyte abnormalities. A third aim was to describe the utility of circulating aldosterone concentrations, if measured, in order to demonstrate mineralocorticoid production status in those dogs presenting without electrolyte abnormalities.

## Methods

### Case selection

Case records of dogs with naturally occurring hypoadrenocorticism presenting to the University College Dublin (UCD) Veterinary Hospital between June 2000 and October 2023 were retrospectively reviewed. Dogs were excluded if there was a prior history (within 6 weeks) of chronic glucocorticoid or mineralocorticoid therapy or where case records were incomplete. Dogs were included if they had evidence of glucocorticoid deficiency, defined as inadequate cortisol production during an adrenocorticotropic hormone (ACTH) stimulation test (post ACTH cortisol < 55 nmol/L or lack of stimulation if > 55 nmol/L with other supportive evidence of hypoadrenocorticism). Cases that had received a dexamethasone injection within one week of presentation were included if aldosterone concentrations were measured and there were other supportive clinicopathological findings. Dogs were then separated into two groups based on circulating sodium and potassium concentrations: dogs with primary hypoadrenocorticism with electrolyte abnormalities (Group 1) and those with EEH (Group 2).

### Clinicopathological evaluation

Blood samples were obtained by jugular venipuncture and placed in EDTA or lithium heparin tubes for haematological and biochemical analyses, respectively. All analyses were performed at the UCD Veterinary Diagnostic Laboratory within 3 to 4 h of collection using the CELL-DYN 3500R (Abbott Laboratories) (up to 2005) or Advia 2120 (Siemens Medical Solutions Diagnostics) (from 2005) haematology analysers and the RX Imola (Randox) (up to 2019) and Attelica (Siemens Medical Solutions Diagnostics) (from 2019) biochemistry analysers. Electrolyte concentrations, including ionised calcium, were also measured in heparinised blood samples immediately after collection using the Rapidpoint 500 (Siemens Medical Solutions Diagnostics).

ACTH stimulation tests were performed in a standardized manner. Cortisol concentrations were measured before and 30 to 60 min after administration of tetracosactide (Synacthen, Alliance or Tetracosactide Solution for Injection/Cosacthen, Dechra Veterinary Products, depending on availability) using a chemiluminescent assay (Immulite 1000 (up to 2012) or 2000 (from 2012), Siemens Medical Solutions Diagnostics). Samples were also analysed in external commercial veterinary laboratories if required (typically Axiom, IDEXX and Veterinary Laboratory Services Ireland). Tetracosactide was administered intravenously at a dose of 250 ug for dogs > 5 kg and 125 ug for dogs < 5 kg or at 5 ug/kg. Basal and ACTH-stimulated aldosterone, and endogenous ACTH concentrations were measured by validated radioimmuno- and enzyme linked immunosorbent assays, respectively, at NationWide Laboratories, UK.

### Data analyses

Signalment, clinical presentation, clinicopathological data and follow-up when available, were retrieved. Considering the different clinicopathological analysers used during the study period, the various parameters were categorised as within, above or below their respective reference intervals. The most common breeds, clinical signs and clinicopathological abnormalities were compared between the two groups. Continuous data were assessed for normality using the Shapiro-Wilk method to direct subsequent selection of statistical tests. Data are reported as median and interquartile range (IQR) or mean ± standard deviation (sd) as appropriate. Categorical data were compared using a χ^2^ test. Continuous data were compared using a Mann-Whitney U-test. Hormone concentrations below the limit of detection of the assays were assigned a value at the limit of detection for statistical analyses. Dogs were diagnosed as aldosterone deficient if the post ACTH aldosterone concentration was undetectable or below reference interval. The utility of basal aldosterone concentrations in predicting aldosterone deficiency was assessed by calculating the positive predictive value (dividing the number of dogs with aldosterone deficiency by the number of dogs that had basal aldosterone concentrations that were undetectable, irrespective of post ACTH concentrations). Statistical significance was set at *p* < 0.05. Statistical analysis was performed using SPSS version 29.0 (IBM).

## Results

### Population

Ninety-two dogs diagnosed with glucocorticoid deficiency associated with naturally occurring hypoadrenocorticism were identified over the study period. They included 72 (78.3%) dogs in Group 1 and 20 (21.7%) dogs in Group 2. Thirty-nine (54.2%) dogs in Group 1 had both hyperkalaemia and hyponatraemia, 29 (40.3%) hyponatraemia only, and 4 (5.5%) hyperkalaemia only. None of the dogs in Group 2 had a sodium: potassium ratio < 27.

In Group 2, three (15.0%) dogs were diagnosed with secondary hypoadrenocorticism. All had low endogenous ACTH concentrations (< 5 pg/mL in two cases and 11 pg/mL in one case; reference interval 5–45 pg/mL) and two of them had pituitary macroadenomas based on magnetic resonance imaging (MRI). Of the remaining 17 (85.0%) cases, endogenous ACTH was measured in five and was increased in each (> 600, > 500, 427, 159 and 48 pg/mL) suggestive of primary hypoadrenocorticism.

Median age was 5.0 (3.0–6.0) years. Dogs in Group 1 were significantly (*p* = 0.001) younger (4.0 (3.0–6.0) years) than dogs in Group 2 (6.0 (4.75–8.25) years). There were more females (*n* = 58 (18 entire, 40 neutered), 63.0%) than males (*n* = 34 (10 entire, 24 neutered, 37.0%), but sex distribution was not significantly different (*p* = 0.750) between the two groups. There were more pedigree dogs (*n* = 65, 70.7%) than crossbreeds (*n* = 27, 29.3%) with no significant difference (*p* = 0.643) between the two groups. Twenty-nine different breeds were represented with cocker spaniels and WHWTs being most common (Table 1). Six of the crossbreeds were poodle crosses (cockapoo (*n* = 2), labradoodle (*n* = 2), golden doodle (*n* = 1) and cavapoo (*n* = 1)) and there was one pedigree standard poodle.

**Table 1:** The most frequent breeds in 92 dogs with hypoadrenocorticism including 72 dogs with (Group 1) and 20 dogs without (Group 2) electrolyte abnormalities. Of the remaining dogs, there was one each of 20 different breeds

	Group 2 No. (%)	Group 1 No. (%)
Crossbreed	23 (31.9)	5 (25.0)
Cocker spaniel	16 (22.2)	1 (5.0)
West Highland white terrier	5 (6.9)	3 (15.0)
Bichon frisé	3 (4.2)	1 (5.0)
Weimaraner	2 (2.8)	0 (0)
Samoyed	2 (2.8)	1 (5.0)
Labrador retriever	1 (1.4)	1 (5.0)
Jack Russel terrier	1 (1.4)	3 (15.0)
Bassett hound	1 (1.4)	2 (10.0)
Cavalier King Charles spaniel	1 (1.4)	1 (1.4)

### Clinical presentation

Time from onset of clinical signs to presentation was available for 56 dogs. Median time from onset of clinical signs to presentation was 14 (7–28) days and did not differ significantly (*p* = 0.119) between Group 1 (14 (7–21) days) and Group 2 (28 (17.5–134.8) days). Clinical signs at presentation were available for 91 dogs (71 dogs in Group 1 and 20 dogs in Group 2) and are presented in Table 2. Only vomiting was significantly (*p* < 0.001) more common in dogs in Group 1.

**Table 2:** Presenting clinical features in 91 dogs with hypoadrenocorticism including 71 dogs with (Group 1) and 20 dogs without (Group 2) electrolyte abnormalities

	All dogs	Group 1	Group 2	*P* value
	No. (%)	No. (%)	No. (%)	
**Lethargy**	64 (71.1)	51 (71.8)	13 (65.0)	0.555
**Vomiting**	58 (64.4)	52 (73.2)	6 (30.0)	< 0.001
**Anorexia/hyporexia**	51 (56.7)	43 (60.6)	8 (40.0)	0.102
**Diarrhoea**	18 (20.0)	16 (22.5)	2 (10.0)	0.214
**Weight loss**	12 (13.3)	9 (12.7)	3 (15.0)	0.786
**Abdominal pain**	12 (13.3)	7 (9.9)	5 (25.0)	0.077
**Collapse**	11 (12.2)	7 (9.9)	4 (20.0)	0.219
**Polyuria/polydipsia**	12 (13.3)	8 (11.3)	4 (20.0)	0.308
**Melaena**	10 (11.1)	9 (12.7)	1 (5.0)	0.332
**Weakness**	9 (10.0)	6 (8.5)	3 (15.0)	
**Trembling**	8 (8.9)	7 (9.9)	1 (5.0)	
**Exercise intolerance**	6 (6.7)	4 (5.6)	2 (10.0)	
**Haematochezia**	6 (6.7)	4 (5.6)	2 (10.0)	
**Haematemesis**	3 (3.3)	3 (4.2)	0 (0.0)	
**Panting**	2 (2.2)	2 (2.8)	0 (0.0)	
**Regurgitation**	2 (2.2)	0 (0.0)	2 (10.0)	
**Dyschezia**	2 (2.2)	2 (2.8)	0 (0.0)	
**Flatulence**	1 (1.1)	0 (0.0)	1 (5.0)	
**Gagging**	1 (1.1)	1 (1.4)	0 (0.0)	
**Hypersalivation**	1 (1.1)	0 (0.0)	1 (5.0)	
**Lip smacking**	1 (1.1)	0 (0.0)	1 (5.0)	
**Listlessness**	1 (1.1)	1 (1.4)	0 (0.0)	

### Clinicopathological abnormalities

Clinicopathological abnormalities are presented in Table 3. Reticulocytosis was significantly (*p* = 0.023) more common in dogs in Group 2 and increased total protein, creatinine, urea and phosphate concentrations were significantly (*p* = 0.023, *p* = 0.021, *p* < 0.001, *p* < 0.001, respectively) more common in dogs in Group 1. There were no other significantly different abnormalities.

**Table 3:** Number of clinicopathological abnormalities at presentation in 92 dogs with hypoadrenocorticism including 72 dogs with (Group 1) and 20 dogs without (Group 2) electrolyte abnormalities

	All dogs		Group 1		Group 2		*P* value
	No. of available case data	No. (%)	No. of available case data	No. (%)	No. of available case data	No. (%)	
Increased haematocrit	92	15 (16.3)	72	14 (19.4)	20	1 (5.0)	0.121
Decreased haematocrit	92	23 (25.0)	72	18 (25.0)	20	5 (25.0)	1
Increased haemoglobin	92	24 (26.1)	72	22 (30.6)	20	2 (10.0)	0.064
Decreased haemoglobin	92	13 (14.1)	72	11 (15.3)	20	2 (10.0)	0.549
Increased red blood cell count	92	17 (18.5)	72	13 (18.1)	20	4 (20.0)	0.843
Decreased red blood cell count	92	17 (18.5)	72	13 (18.1)	20	4 (20.0)	0.843
Reticulocytosis	51	9 (17.6)	38	4 (10.5)	13	5 (38.5)	0.023
Leukocytosis	92	36 (39.1)	72	30 (41.7)	20	6 (30.0)	0.344
Neutrophilia	92	31 (33.7)	72	26 (36.1)	20	5 (25.0)	0.559
Lymphocytosis	92	25 (27.2)	72	19 (26.4)	20	6 (30.0)	0.748
Eosinophilia	84	12 (14.3)	64	8 (12.5)	20	4 (20.0)	0.403
Monocytosis	92	14 (16.3)	72	13 (18.1)	20	2 (10.0)	0.388
Total hypoproteinaemia	91	18 (19.8)	71	11 (15.5)	20	7 (35.0)	0.053
Total hyperproteinaemia	91	22 (24.2)	71	21 (29.6)	20	1 (5.0)	0.023
Hypoalbuminaemia	92	19 (20.7)	72	12 (16.7)	20	7 (35.0)	0.149
Hyperalbuminaemia	92	20 (21.7)	72	17 (23.6)	20	3 (15.0)	0.409
Hypoglobulinaemia	83	24 (28.9)	63	19 (30.2)	20	5 (25.0)	0.657
Hyperglobulinaemia	83	3 (3.6)	63	1 (1.6)	20	2 (20.0)	0.079
Total hypercalcaemia	92	30 (32.6)	72	27 (36.1)	20	3 (15.0)	0.072
Ionised hypercalcaemia	66	10 (15.2)	59	8 (13.6)	7	2 (28.6)	0.295
Hypoglycaemia	92	12 (13.0)	72	10 (13.9)	20	2 (10.0)	0.648
Increased urea	92	57 (62.0)	72	52 (72.2)	20	5 (25.0)	< 0.001
Increased creatinine	92	34 (37.0)	72	31 (43.1)	20	3 (15.0)	0.021
Hypocholesterolaemia	92	30 (32.6)	72	23 (31.9)	20	7 (35.0)	0.797
Increased alkaline phosphatase	92	70 (76.1)	72	54 (75.0)	20	16 (80.0)	0.643
Increased gamma-glutamyl transferase	66	2 (3.0)	51	0 (0)	15	2 (13.3)	ND
Increased alanine transaminase	92	69 (75.0)	72	53 (73.6)	20	16 (80.0)	0.559
Increased aspartate aminotransferase	61	33 (54.1)	47	23 (48.9)	14	10 (71.4)	0.138
Increased creatine kinase	88	71 (80.7)	68	54 (79.4)	20	17 (85.0)	0.578
Hyperbilirubinaemia	90	6 (6.7)	70	4 (5.7)	20	2 (10.0)	0.498
Hyperphosphataemia	91	42 (46.2)	71	40 (56.3)	20	2 (10.0)	< 0.001

### Basal and post-ACTH cortisol and aldosterone concentrations at presentation

Pre and post ACTH cortisol concentrations were all undetectable in the 9 samples sent to external laboratories. Basal cortisol concentrations were measured in-house by chemiluminescent assay in the remaining 83 cases. Concentrations were less than the lowest standard (27.6 nmol/L) in 76 (91.6%) and less than 55.0 nmol/L in 80 (96.4%) dogs. Post ACTH cortisol concentrations were less than 27.6 nmol/L in 75 (93.8%), and less than 55 nmol/L in 78 (97.5%) dogs. Three dogs had undetectable basal cortisol concentrations with post ACTH cortisol concentrations of 57.9, 81 and 149 nmol/L, respectively. The former dog was diagnosed with typical primary hypoadrenocorticism. The second dog had secondary disease with undetectable endogenous ACTH concentration and a large pituitary macroadenoma. The third dog presented in an Addisonian crisis and had just started a hydrocortisone infusion as the post ACTH sample was taken. The three dogs with basal cortisol concentrations greater than 55 nmol/L had values of 130, 92.7 and 62.1, with post ACTH cortisol concentrations of 120, 102.6 and 84.4 nmol/L, respectively. The first dog had a post-mortem examination demonstrating bilateral adrenal destruction from disseminated metastatic anaplastic carcinoma. The second dog had an appropriate treatment response to an Addisonian crisis and the third dog had undetectable aldosterone concentrations pre and post ACTH administration.

In total, forty-four dogs had available basal (reference interval < 20–393 pmol/L) and ACTH-stimulated (reference interval 82–859 pmol/L) aldosterone concentrations at admission, including 30 (41.7%) dogs in Group 1 and 15 (75.0%) dogs in Group 2. All of the dogs in Group 1 had undetectable (< 20 pmol/L) basal and post-ACTH aldosterone concentrations. This included 5 dogs that had received a dexamethasone injection within 7 days of presentation, all of which had corresponding cortisol concentrations < 27.6 nmol/L. Basal aldosterone concentration was undetectable for 5 (33.3%) dogs in Group 2 and detectable (241.3 ± 175.6 pmol/L) in the remaining 10 (66.7%) dogs. ACTH-stimulated aldosterone concentration remained undetectable or below reference interval (50 pmol/L) in 3 of the 5 dogs with undetectable basal aldosterone concentration and increased to within reference interval in the remaining two dogs (403 and 458 pmol/L). One of these dogs was known to have secondary hypoadrenocorticism and the other had an increased endogenous ACTH concentration of 48 pg/mL. Overall, undetectable basal aldosterone concentrations as a predictor of undetectable or below reference interval post ACTH concentrations had a diagnostic test positive predictive value of 94.3%.

Of the remaining 10 dogs in Group 2, one had a post ACTH aldosterone concentration near the lower reference limit (pre and post ACTH concentration 34 and 83 pmol/L, respectively) and was considered aldosterone deficient. In the remaining 9 dogs, post ACTH aldosterone concentrations ranged from 230 to 913 pmol/L (486.6 ± 240.5 pmol/L), including one dog with secondary hypoadrenocorticism. In the 11 dogs that had reference interval post ACTH aldosterone concentrations, there was a median increase of 2.2 (1.5–3.5) times over basal values. In one of these dogs the basal aldosterone concentration (358 pmol/L) did not increase after ACTH administration (315 pmol/L). This dog had adrenal destruction from metastatic neoplasia at post-mortem examination as described above. The two dogs with secondary hypoadrenocorticism had two of the highest increments of 5.7 and a 20.2 from basal to post ACTH aldosterone concentrations. The third highest increment was from a dog that had undetectable basal aldosterone and a post ACTH aldosterone concentration within reference interval (458 pmol/L) for a 22.9-fold increase. This dog had an increased endogenous ACTH concentration at 48 pg/mL. Overall, there was no significant difference (*p* = 0.175) in the aldosterone increment between the two dogs with known secondary hypoadrenocorticism and the remaining 9 dogs.

### Follow-up of dogs with eunatraemic, eukalaemic hypoadrenocorticism

Excluding dogs with known secondary hypoadrenocorticism (*n* = 3), and those euthanased at presentation because of metastatic neoplasia or concurrent haemangiosarcoma (*n* = 1 each), lost to follow-up (*n* = 3) or treated with desoxycorticosterone pivalate (DOCP, Zycortal, Dechra Veterinary Products) at presentation because of aldosterone deficiency (*n* = 3), there were 9 dogs that had longer-term follow-up (median 1,260 (913–1,702) days) (Table 4). Four (44.4%) progressed to have electrolyte abnormalities (*n* = 3) or aldosterone deficiency without electrolyte abnormalities (*n* = 1) within a median of 1,168 (399–1904) days. One of these dogs had undetectable pre and post ACTH aldosterone concentrations at presentation and developed an Addisonian crisis with hyperkalaemia and eunatraemia two months later. Two dogs developed electrolyte abnormalities at 1.4 and 5 years after diagnosis; one presented with hyponatraemia and eukalaemia, and one with hyponatraemia and hyperkalaemia, respectively. Both of these dogs had maintained reference interval post ACTH aldosterone concentrations approximately 4 and 14 months prior to the development of electrolyte disturbances, although with a gradual decline in aldosterone values over time in one dog. One dog was documented to be aldosterone-deficient after 5.9 years despite remaining eunatraemic and eukalaemic. Of the remaining dogs, three had no electrolyte changes during available follow-up. These dogs had ACTH-stimulated aldosterone concentrations that remained within reference interval 3.5, 7.0 and 36 months after diagnosis. One further dog was euthanized after 3.5 years by the referring veterinary practice because of hindlimb paresis without any further information. The remaining dog was alive 2.2 years later without any reported signs of progression.

**Table 4:** Long-term follow-up in 9 dogs with eunatraemic, eukalaemic hypoadrenocorticism (Group 2) and follow-up basal aldosterone (top value) and post-ACTH aldosterone (bottom value) concentrations in pmol/L

Case number	Values on admission	1st follow-up (days)	1st follow-up values	2nd follow-up (days)	2nd follow-up values	3rd follow-up (days)	3rd follow-up values	4th follow-up (days)	4th follow-up values	5th follow-up (days)	5th follow-up values	Progressed to typical (time of progression)
**Case 3**	299 379	1608	< 20 321	1702	< 20 197							Yes − 5 years (developed hyponatraemia and hyperkalaemia)
**Case 4**	< 20 < 20	63										Yes – 63 days (developed hyperkalaemia)
**Case 6**	175 486	42	49 808									Yes − 1.4 years (developed hyponatraemia)
**Case 7**	111 230	30	114 244	60	590 416	186	535 607	307	644 606	2142	97 75	Aldosterone-deficient but no electrolyte changes
**Case 8**	365 573	123	24 564	388	168 424	632	< 20 290	1202	137 708			No
**Case 10**	631 805	108	< 20 140									No (reference interval electrolytes at 1 year)
**Case 11**	109 235	198	179 871	913	65 234							No
**Case 12**	< 20 458											No (reference interval electrolytes at 1 year, euthanised 3 years after diagnosis for hindlimb paresis)
**Case 16**	171 443											No (no signs of progression after 814 days)

ACTH, adrenocorticotropic hormone

## Discussion

This retrospective study aimed to provide details on hypoadrenocorticism in dogs from Ireland, to compare those with and without electrolyte abnormalities and to describe basal and post ACTH stimulated aldosterone concentrations in affected dogs in which it was measured.

Overall, the age, breed and sex of affected dogs concurs with previous reports [26, 28, 36]. The dogs varied widely in age from three months to 12 years but the majority were young middle-aged animals as previously described. There were more females than males and the majority were pedigree dogs from breeds such as cocker spaniels, WHWTs and poodle/poodle crosses that are known to be predisposed to the disease [15]. It is known that a female predisposition is not always present particularly when specific breeds are investigated [7, 19, 20]. However, there were too few dogs of different breeds in the current study to evaluate this further.

The prevalence of EEH in dogs with hypoadrenocorticism of approximately 20% in the present study is higher than older reports of approximately 10% [28] but less than that of over 40% reported more recenly [30]. It likely represents a tendency to specifically test more dogs with a variety of clinical signs in the absence of electrolyte abnormalities because of a growing awareness of its possibility. However, increased utilization of machine-learning methods may enhance diagnostic capability and further increase the diagnosis of this disease as was performed in the study with the highest prevalence [30].

In the present study, dogs with EEH were significantly older than dogs with more typical primary disease as previously reported [16, 25, 34, 35]. The time from onset of clinical signs to presentation did not differ significantly in dogs with or without electrolyte abnormalities. By contrast, another report suggested a more protracted course of disease in dogs with EEH [34]. The difference presumably reflects the wide range in times observed and the tendency to test more dogs more expediently today compared to previously.

The most common clinical features in the cohort of this study included lethargy, various gastrointestinal signs (anorexia/hyporexia, vomiting, diarrhoea, weight loss, abdominal pain, melaena), collapse and polyuria/polydipsia as previously reported [26, 28, 36]. Dogs with typical primary hypoadrenocorticism more commonly presented with vomiting than those with EEH as previously reported [34]. The reason for such a difference between the two groups of dogs is unclear. However, it is possible that the presence of hypovolaemic shock or azotaemia, that is much more common in dogs with typical disease, plays a role in the development of vomiting. No other differences in clinical presentation were identified in this study. Some clinical signs, more typically associated with mineralocorticoid deficiency, such as polyuria and polydipsia, were not statistically different between the two groups. Polyuria and compensatory polydipsia occur as a result of salt wasting associated with mineralocorticoid deficiency and would be expected more commonly in dogs with primary typical disease. However, polyuria and polydipsia have been noted previously in dogs with EEH [25, 34]. Whilst the underlying mechanism of polyuria and polydipsia in dogs with EEH remains unknown and is likely multifactorial, ionised hypercalcaemia resulting from glucocorticoid deficiency could contribute to the polyuria and polydipsia observed in some cases [15]. Owner under-recognition of this clinical sign in more typical cases could also account for the lack of difference seen in this study. Overall, in both types of hypoadrenocorticism, the clinical features are vague and non-specific and frequently involve gastrointestinal signs. Given this, hypoadrenocorticism should be ruled in or out in any dog presenting with gastrointestinal signs before more invasive diagnostic tests are performed [18].

Various clinicopathological abnormalities were observed in this study. The most common haematological abnormalities included leukocytosis, neutrophilia, lymphocytosis and erythrocytosis or anaemia. Anaemia, due to decreased erythropoiesis and/or gastrointestinal haemorrhage, and erythrocytosis, suspected secondary to dehydration, were both identified in this study in just over 15% and approximately 25% of cases, respectively, as has been previously reported [15]. In the face of glucocorticoid deficiency, eosinophilia and lymphocytosis are expected, but eosinophilia and lymphocytosis only occurred in approximately 12 and 25% of the cases, respectively. Of these, over 75% and 65% dogs had eosinophil and lymphocyte count within their respective reference intervals. In the face of systemic illness, this can be considered as suspicious, as eosinopenia and lymphopenia are common responses to illness. This emphasizes the need to consider hypoadrenocorticism in the absence of the expected haematological abnormalities and should not preclude investigation of hypoadrenocorticism [15, 16, 35]. The most common biochemical abnormalities included azotaemia with hyperphosphataemia, various increases in liver enzyme activities and hypocholesterolaemia. The presence of azotaemia is commonly reported in dogs with hypoadrenocorticism as a consequence of mineralocorticoid deficiency. Aldosterone plays a major role in renal sodium reabsorption and its absence contributes to hypovolaemia by allowing increased sodium and water loss. Haemoconcentration and renal hypoperfusion then occur and result in the observed clinicopathological abnormalities. However, fewer dogs in the present study had azotaemia compared to previous reports [15, 28]. Such differences could be explained by the referral nature of the population, as many dogs received intravenous fluid therapy prior to presentation, possibly correcting, at least in part, hypovolaemia and dehydration. Hypocholesterolaemia was common in the present study occurring in approximately one third of the dogs similar to other studies [26, 29, 35]. Hypoadrenocorticism is a common cause of hypocholesterolaemia [14] and is highly discriminatory for dogs with hypoadrenocorticism [29]. On the other hand, the prevalence of hypoglycaemia in this study was lower than previously reported, particularly in dogs with EEH [26, 28, 35]. Many dogs that presented with hypoglycaemia at the referring veterinarian were supplemented with intravenous dextrose prior to presentation, likely explaining the low prevalence of hypoglycaemia in this study. Hypercalcaemia is reported in up to a third of dogs with hypoadrenocorticism [15, 16] This is similar to the present study, and although there was a lower prevalence of ionised hypercalcaemia, the existence of total without ionised hypercalcaemia is recognized [16]. The underlying mechanisms for hypercalcaemia remain poorly understood but presumably relate in part to glucocorticoid deficiency and reduced calciuresis but are clearly influenced by hyperalbuminaemia and potentially complexed calcium concentrations and acid base status. The more common increase in serum total protein, urea, creatinine, and phosphorus concentrations in dogs with mineralocorticoid deficiency compared to those without electrolyte abnormalities in the current study has been reported previously and aligns with the expected physiological consequences of mineralocorticoid deficiency [16, 34]. Interestingly, the frequency of reticulocytosis in dogs with EEH has not been documented previously. While the low number of dogs with reticulocytosis makes interpretation difficult, its finding could suggest the potential occurrence of occult gastrointestinal bleeding. Anaemia and hypoalbuminaemia were more common findings in dogs with EEH in previous reports suggesting that gastrointestinal haemorrhage is important in this disease [34]. Glucocorticoids play a role in maintaining gastrointestinal mucosal integrity and motility. The current study indicates that the presence of unexplained reticulocytosis should prompt further consideration of hypoadrenocorticism as a potential underlying cause.

In the present study, the results of the ACTH stimulation test were used to diagnose hypoadrenocorticism through demonstration of diminished cortisol reserve. Over 95% of dogs had basal cortisol concentrations ≤ 55 nmol/L, a value suggested to have a diagnostic test sensitivity of over 99% [5, 6, 12, 24]. The fact that approximately 5% of dogs in the present study had basal values greater than this, suggests tht these dogs may be misdiagnosed if such a cut-off is solely relied upon. The three dogs with higher basal cortisol values demonstrated no stimulation of cortisol after administration of exogenous ACTH confirming diminished cortisol reserve. One of these dogs had adrenal destruction from neoplastic invasion, an unusual form of hypoadrenocorticism [15]. The two remaining dogs presented with Addisonian crises and had appropriate and rapid responses to fluid and hydrocortisone therapy. One of these dogs had undetectable pre and post ACTH aldosterone concentrations, confirming the diagnosis. In three other dogs, there was apparent stimulation of cortisol from undetectable concentrations to values greater than 55 nmol/L, again a cut-off usually considered as diagnostic of hypoadrenocorticism [15]. One dog had central disease, a known pituitary macroadenoma and undetectable endogenous ACTH concentration and was specifically being evaluated for cortisol deficiency on this basis. One dog had a post ACTH value close to the diagnostic cut-off. The remaining dog had just started hydrocortisone therapy accounting for the apparent stimulation and exemplifies the need to assess cortisol concentrations prior to commencing such therapy. Nevertheless, it had an appropriate response to fluid and hydrocortisone therapy and required mineralocorticoid replacement for the remainder of its life.

In the present study, aldosterone concentrations were measured to confirm hypoadrenocorticism in dogs if prior glucocorticoid administration was a possibility and to assess aldosterone reserve in those dogs without electrolyte abnormalities. The results suggest that such measurement may be valuable in dogs presenting with hypoadrenocorticism. Firstly, basal and post ACTH values, when measured, were all below reference interval in dogs presenting with typical disease, including the five dogs that had recently received dexamethasone. This suggests some value in confirming a diagnosis if cortisol concentrations are potentially affected by prior treatment or are equivocal. In some dogs with EEH in the current study, aldosterone deficiency was confirmed at presentation or over time. The fact that electrolyte concentrations can remain within reference interval despite aldosterone deficiency has been reported previously and may represent an as of yet unknown mechanism [2]. It could also represent prior symptomatic treatment, such as intravenous fluid therapy, as this may correct electrolyte abnormalities prior to presentation [28]. There is controversy regarding the need for treatment with mineralocorticoids in these dogs. In this study, one dog in which such treatment was not expediently introduced, developed an Addisonian crisis within two months, a rapid progression of disease reported previously [34]. There are few studies specifically evaluating progression of disease in dogs with EEH. In one study, three dogs with EEH and aldosterone deficiency were followed up; one was pre-emptively treated with mineralocorticoids, one developed hyperkalaemia within 6 months and one maintained eukalaemia and eunatraemia over a two-year period [2]. Electrolyte abnormalities did not develop in another study of 12 dogs but they were only followed up for a median of 284 days and aldosterone concentrations were not measured in any [33]. Certainly, the results presented here suggest that if aldosterone concentrations are initially adequate, deficiency as assessed by development of electrolyte abnormalities or supportive aldosterone concentrations can and do occur, although it can take years to develop. Overall, the demonstration of clear aldosterone deficiency provides some evidence to consider mineralocorticoid supplementation as was done in the current study. This could be taken into consideration with other factors such as the possibility of prior treatment influencing electrolyte concentrations, the ability of the owner to expediently recognise signs of an impending crisis, and the cost and availability of emergency treatment if required. Consideration may also be given to the growing body of human research on extra-renal effects of mineralocorticoids and the existence of aldosterone sensitive neurons within the brain that may be important in mood and behaviour [11, 27]. Evaluation of aldosterone concentrations may also be used to guide the necessity for more intense (aldosterone deficient dogs) or less intense (aldosterone sufficient dogs) monitoring practices.

In practice, the decision to measure aldosterone concentrations is often dictated by the cortisol results. Sufficient sample volume is a requirement but may not always be available. In the present study an undetectable basal aldosterone concentration was highly predictive of an undetectable post ACTH value, suggesting that basal values alone may provide support for aldosterone deficiency, if post ACTH samples are not available. The median increase in aldosterone concentrations after ACTH administration was two-fold in dogs with EEH. Notably, although not statistically significant, two of the three highest increments (5.7 and 20.2) occurred in dogs with secondary hypoadrenocorticism compared to the majority of dogs displaying increases ranging from 0.8 to 2.8 times. This observation implies a potential disparity in the aldosterone increment following ACTH administration in dogs with documented secondary hypoadrenocorticism. Only one other dog exhibited an apparently exaggerated aldosterone response (22.9-fold increase). Although endogenous ACTH concentration was mildly increased in this dog further investigations were not carried out over time. The possibility that a diagnosis of secondary hypoadrenocorticism be prompted by the extent of change in aldosterone concentrations during an ACTH stimulation test requires further investigation.

The present study had many limitations. Firstly, its retrospective nature, which resulted in the lack of long-term follow-up, and missing data in some cases. Secondly, the use of different analyzers and reference intervals during the study period limited the statistical analysis of haematological and biochemical data. Finally, the lack of endogenous ACTH concentration measurement in all dogs with EEH limits the differentiation of primary and secondary disease.

## Conclusion

Dogs with hypoadrenocorticism from Ireland resemble those previously reported. A relevant proportion of dogs present without electrolyte abnormalities and are challenging diagnostically because of their vague signs and lack of pathognomonic clinicopathological features. Measurement of basal and ACTH stimulated aldosterone concentrations is important as some of these dogs have concurrent mineralocorticoid deficiency despite remaining eunatraemic and eukalaemic. An undetectable basal aldosterone concentration is a reasonable predictor of mineralocorticoid deficiency. Exaggerated stimulation of aldosterone secretion following ACTH administration may indicate a need to investigate for secondary hypoadrenocorticism. Further studies are required to assess progression of disease particularly in dogs with EEH and demonstrable aldosterone deficiency.

## Data Availability

The datasets analysed during the current study are available from the corresponding author on reasonable request.

## Abbreviations

ACTH: Adrenocorticotropic hormone
EEH: Eunatraemic, eukalaemic hypoadrenocorticism
DOCP: Deoxycorticosterone pivalate
GPMD: Great Pyrenean Mountain dogs
MRI: Magnetic resonance imaging
NSDTR: Nova Scotia duck tolling retrievers
SCWT: Soft-coated wheaten terriers
UCD: University College Dublin
WHWT: West Highland white terrier
